# Application of the HIV prevention cascade to identify, develop and evaluate interventions to improve use of prevention methods: examples from a study in east Zimbabwe

**DOI:** 10.1002/jia2.25309

**Published:** 2019-07-22

**Authors:** Louisa Moorhouse, Robin Schaefer, Ranjeeta Thomas, Constance Nyamukapa, Morten Skovdal, Timothy B Hallett, Simon Gregson

**Affiliations:** ^1^ Department of Infectious Disease Epidemiology MRC Centre for Global Infectious Disease Analysis Imperial College London London UK; ^2^ Department of Health Policy London School of Economics and Political Science London UK; ^3^ Biomedical Research and Training Institute Harare Zimbabwe; ^4^ Department of Public Health University of Copenhagen Copenhagen Denmark

**Keywords:** HIV prevention cascade, HIV prevention interventions, adolescent girls and young women, young men, Zimbabwe

## Abstract

**Introduction:**

The HIV prevention cascade could be used in developing interventions to strengthen implementation of efficacious HIV prevention methods, but its practical utility needs to be demonstrated. We propose a standardized approach to using the cascade to guide identification and evaluation of interventions and demonstrate its feasibility for this purpose through a project to develop interventions to improve HIV prevention methods use by adolescent girls and young women (AGYW) and potential male partners in east Zimbabwe.

**Discussion:**

We propose a six‐step approach to using a published generic HIV prevention cascade formulation to develop interventions to increase motivation to use, access to and effective use of an HIV prevention method. These steps are as follows: (1) measure the HIV prevention cascade for the chosen population and method; (2) identify gaps in the cascade; (3) identify explanatory factors (barriers) contributing to observed gaps; (4) review literature to identify relevant theoretical frameworks and interventions; (5) tailor interventions to the local context; and (6) implement and evaluate the interventions using the cascade steps and explanatory factors as outcome indicators in the evaluation design. In the Zimbabwe example, steps 1‐5 aided development of four interventions to overcome barriers to effective use of pre‐exposure prophylaxis (PrEP) in AGYW (15‐24 years) and voluntary medical male circumcision in male partners (15‐29). For young men, prevention cascade analyses identified gaps in motivation and access as barriers to voluntary medical male circumcision uptake, so an intervention was designed including financial incentives and an education session. For AGYW, gaps in motivation (particularly lack of risk perception) and access were identified as barriers to PrEP uptake: an interactive counselling game was developed addressing these barriers. A text messaging intervention was developed to improve PrEP adherence among AGYW, addressing reasons underlying lack of effective PrEP use through improving the capacity (“skills”) to take PrEP effectively. A community‐led intervention (community conversations) was developed addressing community‐level factors underlying gaps in motivation, access and effective use. These interventions are being evaluated currently using outcomes from the HIV prevention cascade (step 6).

**Conclusions:**

The prevention cascade can guide development and evaluation of interventions to strengthen implementation of HIV prevention methods by following the proposed process.

## 1 Introduction

HIV prevention cascades may facilitate identification and understanding of gaps in use of primary HIV prevention methods and identification and evaluation of interventions to address the gaps 1, 2, 3, 4.

While HIV treatment cascades – describing the steps required to achieve viral suppression 5 – have aided design of interventions to improve treatment programmes (e.g. in Uganda 6) and cascades are utilized in prevention of mother‐to‐child HIV transmission programmes 7, formulations of the HIV prevention cascade have been largely theoretical and its utility in identifying appropriate interventions remains to be demonstrated 1, 2, 3, 4, 8, 9, 10, 11, 12, 13, 14. We present a standardized approach to using the prevention cascade to guide identification, development and evaluation of interventions to increase effective use of HIV prevention methods. We demonstrate the feasibility of this approach by describing the development and pilot testing of interventions to reduce HIV risk among adolescent girls and young women (AGYW) in Manicaland, east Zimbabwe – moving prevention cascades from theory into practice.

## 2 Discussion

### 2.1 HIV prevention cascade framework

**Figure 1:**
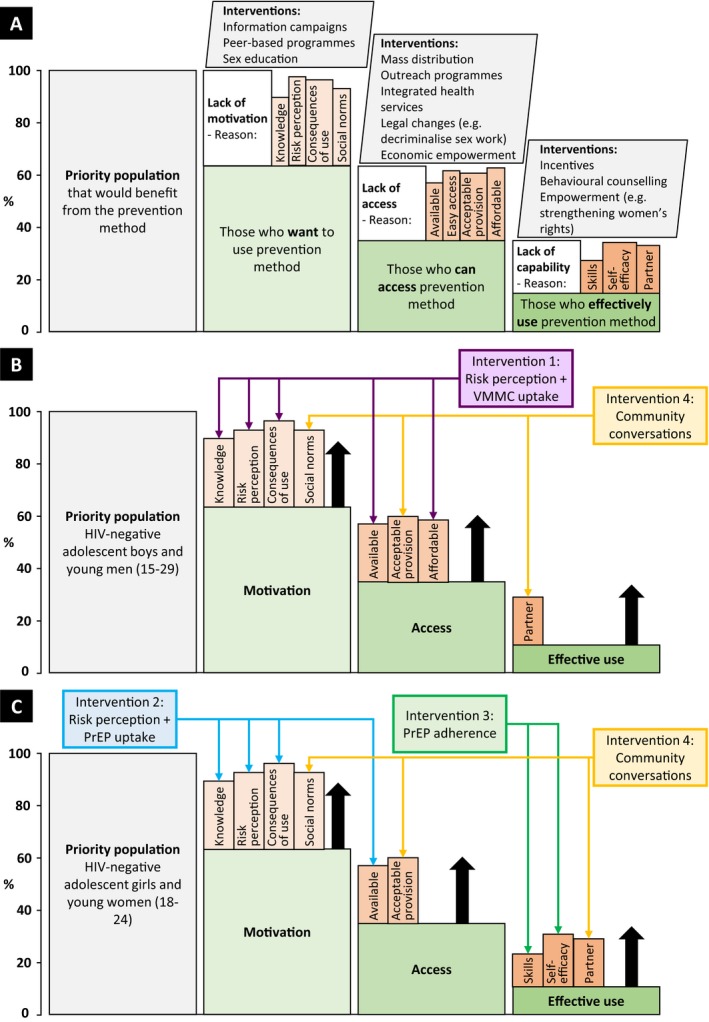
**The generic HIV prevention cascade framework used in this study (A) to guide the design of interventions to improve HIV prevention use among adolescent boys and young men (15‐29) (B) and adolescent girls and young women (18‐24) (C)** The generic prevention cascade includes the core steps of the cascade (green) and the major reasons underlying gaps in the core steps (orange), which provide links to interventions to improve motivation, access, and effective use. The specific interventions referred to in (A,B) are an education session by a circumcised health worker and financial incentives to take up voluntary medical male circumcision (VMMC) (intervention 1), an interactive counselling game to improve risk perception and pre‐exposure prophylaxis (PrEP) uptake (intervention 2), weekly text message reminders to improve PrEP adherence (intervention 3), and community conversation (intervention 4).

Our preferred HIV prevention cascade framework – developed through multiple consultations 15 – focuses on three domains for prevention method use in a priority at‐risk population: motivation, access and effective use (Figure 1A). Effective use is the uptake and adherence required to achieve close to the maximum level of protection against HIV infection afforded by the method. The gap between access and effective use reflects lack of capacity to use the method effectively. Justification of this framework is available 4. Key features and advantages are as follows: (1) it is generic so can be applied for any primary prevention method(s) or population; (2) effective use – the endpoint of the cascade – is closely aligned with impact (HIV infections averted); (3) it provides a simple core cascade for high‐level monitoring and advocacy; (4) ease of application to combination HIV prevention; and (5) it reflects that multiple barriers work together limiting effective use of HIV prevention methods.

### 2.2 Standard approach to using the HIV prevention cascade to develop interventions

We propose a series of steps to be followed in using the HIV prevention cascade to identify and evaluate potentially effective interventions to improve use of HIV prevention methods and reduce HIV incidence in an at‐risk population:

1. Measure the HIV prevention cascade for the chosen population and method(s)
2. Examine the three steps in the cascade to identify gaps in use of the method(s), thereby identifying broad targets for interventions
3. Use the best available data on the factors contributing to the gaps identified in the cascade (the sub‐bars in Figure 1A) to establish which specific barriers should be targeted to increase effective use of the method(s)
4. Review the literature to identify theoretical frameworks and interventions that have potential to reduce the factors identified as barriers to effective use
5. Tailor the interventions to the local epidemiological and socio‐economic context
6. Implement the interventions, including the steps and explanatory factors in the HIV prevention cascade as outcomes in the evaluation design

The prevention cascade supports the identification of intervention targets but does not impose specific intervention designs.

### 2.3 Practical examples from Manicaland

#### 2.3.1 Setting and epidemiological context

The HIV prevention cascade is guiding research to develop interventions to reduce HIV incidence in AGYW in Manicaland by increasing effective use of HIV prevention methods including voluntary medical male circumcision (VMMC) in male partners and oral pre‐exposure prophylaxis (PrEP) in AGYW.

Manicaland is a rural 16 province that has been identified as an HIV transmission “hotspot” 17 and is a priority area in the Zimbabwe National HIV and AIDS Strategic Plan 18. Adult (15+) HIV prevalence declined from over 25% in the late 1990s to 11% in 2015‐2016 19. HIV incidence fell from 1.8% in the mid‐2000s to under 1% among females and 0.5% among males 20, partially due to behaviour change 21, 22. HIV prevalence among AGYW (5.4%) in Manicaland is nearly double that of young men (2.9%) 23. HIV incidence among AGYW remains high (1.4% 2009 to 2013) 20. AGYW in Manicaland commonly have sexual relationships with older men while condom use is low 24. Oral PrEP is only available in small‐scale research projects 25. Zimbabwe is a priority country in the DREAMS programme 26. Manicaland is a priority for PrEP introduction for key populations, including AGYW 27. VMMC uptake in Manicaland has been slow 28.

#### 2.3.2 Selection of interventions

The study (Manicaland Study) commenced in July 2018 to identify and test interventions to reduce multi‐level barriers preventing AGYW at risk of HIV infection and potential male partners from using efficacious prevention methods. The study is being implemented in eight sites in Manicaland representing different socio‐economic strata; VMMC and PrEP were selected being relatively new methods of HIV prevention with high efficacy and potential to contribute more to the overall impact of combination prevention in Zimbabwe.

Data collected between 1998 and 2013 in a general‐population cohort study (Manicaland Cohort) 20 were used to measure the preliminary HIV prevention cascades 2, 4 (step 1) and identify gaps in motivation, access, and effective use of PrEP and VMMC in AGYW and their male sexual partners (step 2). Cohort data were analysed to establish which specific barriers to effective use of these methods should be targeted by interventions (step 3). These analyses arranged existing Manicaland Cohort data into this framework. Interventions were identified using behavioural economics and community psychology literature (step 4), and developed and tailored to the local context (step 5) using information from previous studies 24, 29, including qualitative analyses 30, 31. These interventions are being pilot tested using a cluster‐randomized controlled trial design with matched intervention and control clusters in each of the study sites. Indicators based on the HIV prevention cascade are being used as outcomes in this evaluation study. Further details are available at https://clinicaltrials.gov (NCT03565575 and NCT03565588).

The study includes three individual‐based interventions, in eight study sites, and a community‐based intervention implemented in two of these sites. The study addresses individual‐ and community‐level barriers to HIV prevention use – recognizing that HIV prevention behaviour is influenced by multiple factors acting at different levels 32, 33, 34.

##### Intervention 1: Increasing young men's motivation for and access to VMMC

Increasing effective use of VMMC helps to reduce HIV incidence in AGYW by reducing exposure to HIV infection from their male partners. VMMC is central in Zimbabwe's HIV prevention programme, but only 10% of men (15‐49) are circumcised in Manicaland 23 and uptake is off‐track to meet national targets 28.

Previous HIV prevention cascade analysis using Manicaland Cohort data found that low VMMC uptake in the study population was largely due to low risk perception for HIV infection, suggesting gaps in motivation and poor local availability 2. Motivation for VMMC uptake can be affected by negative perceptions of its consequences. Transport costs and lost income were identified as important barriers to access (Figure 1B).

An intervention was developed where HIV‐negative young men (15‐29) participate in an education session on HIV risks and reducing these risks through VMMC, run by a circumcised male “role‐model.” They are randomized to receive a fixed financial reward or the opportunity to participate in a lottery (with financial rewards) upon VMMC uptake. Participants receive a contribution towards transport costs for accessing VMMC and are referred to participating study clinics offering VMMC. The education session aims to increase motivation by improving HIV risk perception, knowledge and perceptions about consequences of VMMC. The financial incentive increases motivation by creating more positive consequences for uptake. Previous behavioural economics research showed lottery tickets may be more effective in increasing motivation than fixed financial rewards as individuals overweight small probability events 35. For access to VMMC, the education session and referral provide information about local availability; the financial incentives and contribution towards transport costs improve its affordability.

Organizing the follow‐up data in the prevention cascade framework aids evaluation by providing data on possible reasons why the intervention may have failed to improve uptake. Changes in the HIV prevention cascade for VMMC will be compared between a baseline and follow‐up survey six months later, for men in the intervention and control groups using HIV risk perception and VMMC uptake as primary outcomes.

##### Intervention 2: Increasing motivation and access for PrEP use among AGYW

No prior measurements of the HIV prevention cascade for PrEP were available for AGYW in the study population. Since PrEP is a new method of HIV prevention in Zimbabwe and is not widely available 25, it was assumed that motivation, access and effective use would be low at the outset.

Earlier analyses of Manicaland Cohort data on risk among AGYW indicated unprotected sexual relationships with older men contribute to their high HIV incidence 24. Preliminary analyses of the HIV prevention cascade for other prevention methods among AGYW found gaps in perceptions about personal HIV infection risks 2, 4. Lack of risk perception indicates lack of motivation to use HIV prevention methods. Evidence from population‐based trials on PrEP in sub‐Saharan Africa suggests low risk perception may limit PrEP use along with doubt about using antiretroviral drugs (ARVs) for prevention 36, 37, 38.

An intervention was designed whereby HIV‐negative AGYW (18‐24) play an interactive counselling game 39, addressing optimistic beliefs about HIV infection risks – particularly around sexual relations with older men – and providing information on the nature, effectiveness, tolerability and local availability of PrEP. Participants can choose to be contacted by a nurse to discuss PrEP further and a referral letter to participating study clinics where PrEP services are available. The intervention aims to adjust HIV risk perception and improve PrEP uptake by targeting barriers in motivation and access to use (Figure 1C). Gaps in motivation are addressed by improving knowledge of the method and consequences of its use and increasing risk perception accuracy. Previous studies showed that providing disaggregated information on HIV risks can adjust risk perception and behaviour 39, 40. Information on and referrals to local PrEP services address gaps in access.

Evaluation will compare the HIV prevention cascades for PrEP for AGYW in the intervention and control groups after six months using HIV risk perception and uptake of PrEP (confirmed by ARV presence in blood samples 41, 42, 43) as primary outcomes.

##### Intervention 3: Increasing effective use of PrEP by AGYW

Effective use of PrEP requires continuous adherence – not just uptake – to provide protection against HIV infection. It is anticipated that this will be a challenge for AGYW 44. AGYW may have difficulties remembering to take PrEP daily and could lack salience about the risks and consequences of HIV infection.

An intervention was designed to improve PrEP adherence among AGYW who are on PrEP whereby they receive unidirectional, personalized text messages, acting as “nudges.” Text messaging “nudges” have been shown to improve ART adherence 45. This intervention addresses the likely gaps in personal capacity to use PrEP effectively, specifically limited salience of HIV risk (Figure 1C), thus improving the “skills” required to adhere consistently. Knowledge of these reminders may improve the self‐perceived ability to adhere to PrEP (self‐efficacy).

This intervention will be evaluated by randomizing AGYW on PrEP into intervention and control groups. Effective use of PrEP (adherence assessed through self‐reports and ARV presence in blood samples) will be compared in the two groups after six months.

##### Intervention 4: Improving social support for young people's use of HIV prevention methods

Some factors contributing to gaps in the cascade lie outside of the individual's control, including influences by partners, peers, families, healthcare providers and social structures 32. Prevention cascade analysis of the study population showed social norms and partner disapproval represent barriers to condom use 4. Individual‐level interventions may have limited impact in increasing effective VMMC and PrEP use if the local social environment is not supportive.

A community conversations (CCs) intervention 46 is being implemented in two study sites to address community‐level barriers contributing to the HIV prevention cascades for VMMC and PrEP. CCs are a community‐led capacity‐building process where community members identify, plan, implement and evaluate their own actions to break down community‐level barriers to engagement with HIV prevention methods. CCs are expected to improve motivation for HIV prevention method use by creating and fostering supportive social norms (Figure 1B,C). Partner approval – an important factor for PrEP use 47 and VMMC 48 – is being addressed. Creating more prevention‐positive social norms in a community may encourage health workers to adopt less stigmatizing attitudes towards HIV prevention use by young people, making provision of HIV prevention services more acceptable and improving access.

Survey and qualitative data will be analysed to evaluate whether the CCs intervention impacted the specific barriers to motivation, access and effective use of PrEP and VMMC by AGYW and young men in the study populations. Prevention cascades will be constructed and compared for VMMC and PrEP in the intervention and control groups between the two CCs sites and the remaining six sites to assess the effectiveness of CCs.

## 3 Conclusions

We have outlined a generic approach to using the HIV prevention cascade to identify, develop and evaluate interventions and demonstrated feasibility of application using the example of a study testing interventions to strengthen implementation of VMMC and PrEP services in Manicaland.

When developing interventions, a central benefit of the HIV prevention cascade framework is that it underscores the multitude of factors to be addressed potentially limiting effective use of prevention methods. As with PrEP in Manicaland, the prevention cascade framework is useful for organizing evidence from other methods and settings to guide thinking about barriers to be addressed in implementing a new method. While the cascade highlights bottlenecks and areas that require interventions to improve progress through the cascade, it does not determine the most suitable interventions – these must be based on theoretical frameworks, local circumstances and evidence from similar settings.

When evaluating interventions, outcomes and process indicators can be defined corresponding to steps and reasons underlying gaps in the HIV prevention cascade, providing a standardized basis to compare intervention and control groups and over time. Cascade analysis can be useful for interpretation of trial results and identifying reasons for the success or failure of interventions. The HIV prevention cascade does not measure the impact of interventions. This is possible – as planned in the Manicaland Study – by mathematical modelling to generate estimates of population‐level impact. The cascade is being used in evaluating the implementation of individual and combination HIV prevention methods to estimate the overall impact of the interventions on HIV incidence.

In the Manicaland Study, the HIV prevention cascade is being measured and interpreted using data from population surveys and qualitative investigations. Other data sources – for example, routinely collected health data – could also be used (see 4 for further discussion).

Evaluation of the aforementioned interventions has not been completed. Nevertheless, the study demonstrates the HIV prevention cascade framework can be used to improve development and evaluation of HIV prevention interventions by setting targets to be addressed to remove bottlenecks in prevention use. The framework can be used for multiple settings, populations and HIV prevention methods as it is generic and adaptable by design, although the risk of stigmatizing specific populations (e.g. AGYW) should be considered. As the HIV treatment cascade has aided a range of policy, programmes and research at multiple levels, we believe this cascade can provide a framework to identify gaps in prevention efforts and targets for interventions. We encourage this approach to inform the intervention development and believe this framework can support global efforts to reduce HIV incidence.

## Competing interests

S.G. declares shareholding in pharmaceutical companies (GSK and Astra Zeneca). R.T. declares personal fees received for consultancy for the International Decision Support Initiative. The authors declare no further potential competing interests.

## Authors’ contributions

All authors have been involved in the design of the Manicaland Study, led by TBH and SG, LM and RS wrote the article, with input from all authors. All authors have read and approved the final manuscript.

## References

[1] Amico KR . Developing an “HIV prevention cascade”: current approaches and future directions. 10th International Conference on HIV Treatment and Prevention Adherence, Miami; 2015.

[2] Garnett GP , Hallett TB , Takaruza A , Hargreaves J , Rhead R , Warren M , et al. Providing a conceptual framework for HIV prevention cascades and assessing feasibility of empirical measurement with data from east Zimbabwe: a case study. Lancet HIV. 2016;3(7):e297–306. 27365204 10.1016/S2352-3018(16)30039-X PMC4935672

[3] Hargreaves JR , Delany‐Moretlwe S , Hallett TB , Johnson S , Kapiga S , Bhattacharjee P , et al. The HIV prevention cascade: integrating theories of epidemiological, behavioural, and social science into programme design and monitoring. Lancet HIV. 2016;3(7):e318–22. 27365206 10.1016/S2352-3018(16)30063-7

[4] Schaefer R , Gregson S , Fearon E , Hensen B , Hallett TB , Hargreaves JR . HIV prevention cascades: a unifying framework to replicate the successes of treatment cascades. Lancet HIV. 2019;6(1):e60–6. 10.1016/s2352-3018(18)30327-8 PMC7025885 32066995

[5] Haber N , Pillay D , Porter K , Bärnighausen T . Constructing the cascade of HIV care: methods for measurement. Curr Opin HIV AIDS. 2016;11(1):102. 26545266 10.1097/COH.0000000000000212

[6] Amanyire G , Semitala FC , Namusobya J , Katuramu R , Kampiire L , Wallenta J , et al. Effects of a multicomponent intervention to streamline initiation of antiretroviral therapy in Africa: a stepped‐wedge cluster‐randomised trial. Lancet HIV. 2016;3(11):e539–48. 27658873 10.1016/S2352-3018(16)30090-X PMC5408866

[7] Hamilton E , Bossiky B , Ditekemena J , Esiru G , Fwamba F , Goga AE , et al. Using the PMTCT cascade to accelerate achievement of the global plan goals. J Acquir Immune Defic Syndr. 2017;75 Suppl 1:S27–35. 28398994 10.1097/QAI.0000000000001325 PMC5400406

[8] San Francisco Department of Public Health . The jurisdictional HIV prevention plans for the San Francisco metropolitan statistical area 2012‐2016. San Francisco: San Francisco Department of Public Health; 2013.

[9] Liu A , Colfax G , Cohen S , Bacon O , Kolber M , Amico KR , et al. The spectrum of engagement in HIV prevention: proposal for a PrEP cascade. 7th International Conference on HIV Treatment and Prevention Adherence, Miami; 2012.

[10] Wilton J , Kain T , Fowler S , Hart TA , Grennan T , Maxwell J , et al. Use of an HIV‐risk screening tool to identify optimal candidates for PrEP scale‐up among men who have sex with men in Toronto, Canada: disconnect between objective and subjective HIV risk. J Int AIDS Soc. 2016;19(1):20777. 27265490 10.7448/IAS.19.1.20777 PMC4911732

[11] Kelley CF , Kahle E , Siegler A , Sanchez T , Del Rio C , Sullivan PS , et al. Applying a PrEP continuum of care for men who have sex with men in Atlanta, Georgia. Clin Infect Dis. 2015;61(10):1590–7. 26270691 10.1093/cid/civ664 PMC4614414

[12] Horn T , Sherwood J , Remien RH , Nash D , Auerbach J , HIV Prevention Continuum Working Group ftTAG . Towards an integrated primary and secondary HIV prevention continuum for the United States: a cyclical process model. J Int AIDS Soc. 2016;19(1):21263. 27863535 10.7448/IAS.19.1.21263 PMC5116064

[13] McNairy ML , El‐Sadr WM . A paradigm shift: focus on the HIV prevention continuum. Clin Infect Dis. 2014;59 Suppl 1:S12–5. 24926026 10.1093/cid/ciu251 PMC4141493

[14] Johnson J . Toward comprehensive HIV prevention service delivery in the United States: an action plan. Washington, DC; New York, NY: The Foundation for AIDS Research; Treatment Action Group; 2015.

[15] Manicaland Centre for Public Health Research . HIV prevention cascades: stakeholder consultation meeting and workshop. 2017 [cited 2018 Oct 30]. Available from: http://www.manicalandhivproject.org/uploads/4/7/1/9/4719905/hpc_consultation_workshop_report_final.pdf.

[16] Zimbabwe National Statistics Agency (ZIMSTAT) . Inter‐censal demographic survey, 2017. Harare: ZIMSTAT; 2017.

[17] Ministry of Healt and Child Care (MOHCC) Zimbabwe, National AIDS Council Zimbabwe, Centers for Disease Control and Prevention USA, UNAIDS . Smart investment to end HIV AIDS in ZIMBABWE based on hotspot analysis. Harare: MOHCC; 2015.

[18] Ministry of Healt and Child Care (MOHCC) Zimbabwe, National AIDS Council Zimbabwe . Extended Zimbabwe national HIV and AIDS strategic plan (ZNASP) 2015‐2020. Harare: MOHCC; 2015.

[19] Ministry of Healt and Child Care (MOHCC) Zimbabwe . Zimbabwe Population‐Based HIV Impact Assessment (ZIMPHIA) 2015‐16: first report. Harare: MOHCC; 2017.

[20] Gregson S , Mugurungi O , Eaton J , Takaruza A , Rhead R , Maswera R , et al. Documenting and explaining the HIV decline in east Zimbabwe: the Manicaland General Population Cohort. BMJ Open. 2017;7(10):e015898. 10.1136/bmjopen-2017-015898 PMC5639985 28988165

[21] Gregson S , Garnett GP , Nyamukapa CA , Hallett TB , Lewis JJ , Mason PR , et al. HIV decline associated with behavior change in eastern Zimbabwe. Science. 2006;311(5761):664–6. 16456081 10.1126/science.1121054

[22] Gregson S , Nyamukapa C , Schumacher C , Mugurungi O , Benedikt C , Mushati P , et al. Did national HIV prevention programs contribute to HIV decline in Eastern Zimbabwe? Evidence from a prospective community survey. Sex Transm Infect. 2011;38(6):475–82. 10.1097/OLQ.0b013e3182080877 PMC3514751 21278627

[23] Zimbabwe National Statistics Agency (ZIMSTAT), ICF International . Zimbabwe Demographic and Health Survey 2015: final report. Rockville, MD: ZIMSTAT and ICF International; 2016.

[24] Schaefer R , Gregson S , Eaton JW , Mugurungi O , Rhead R , Takaruza A , et al. Age‐disparate relationships and HIV incidence in adolescent girls and young women: evidence from Zimbabwe. AIDS. 2017;31(10):1461–70. 28426534 10.1097/QAD.0000000000001506 PMC5457819

[25] Ministry of Health and Child Care Zimbabwe . Implementation plan for HIV pre‐exposure prophylaxis in Zimbabwe 2018‐2020. Harare: Ministry of Health and Child Care Zimbabwe; 2018.

[26] U.S. President's Emergency Plan for AIDS Relief (PEPFAR) . DREAMS partnership: fact sheet. Washington, DC: PEPFAR; 2017.

[27] Pangaea Global . Oral PrEP introduction: Zimbabwe rollout analysis [Online]. 2017 [cited 2018 Oct 7]. Available from: https://www.prepwatch.org/wp-content/uploads/2017/04/Zim_Rollout_Analysis_Feb2017.pdf

[28] Zimbabwe MoHaCCM, National AIDS Council Zimbabwe . Global AIDS response progress report 2017: Zimbabwe Country report. Harare: MOHCC; 2017.

[29] Schaefer R , Thomas R , Nyamukapa C , Maswera R , Kadzura N , Gregson S . Accuracy of HIV Risk Perception in East Zimbabwe 2003‐2013. AIDS Behav. 2018; 10.1007/s10461-018-2374-0. [Epub ahead of print] PMC6647479 30569314

[30] Rhead R , Skovdal M , Takaruza A , Maswera R , Nyamukapa C , Gregson S . The multidimensionality of masculine norms in east Zimbabwe: implications for HIV prevention, testing and treatment. AIDS. 2019;33(3):537–46. 30531399 10.1097/QAD.0000000000002041 PMC6365253

[31] Skovdal M , Campbell C , Madanhire C , Mupambireyi Z , Nyamukapa C , Gregson S . Masculinity as a barrier to men's use of HIV services in Zimbabwe. Global Health. 2011;7:13. 21575149 10.1186/1744-8603-7-13 PMC3107786

[32] Kaufman MR , Cornish F , Zimmerman RS , Johnson BT . Health behavior change models for HIV prevention and AIDS care: practical recommendations for a multi‐level approach. J Acquir Immune Defic Syndr. 2014;66 Suppl 3:S250–8. 25007194 10.1097/QAI.0000000000000236 PMC4536982

[33] Campbell C , Cornish F . Towards a “fourth generation” of approaches to HIV/AIDS management: creating contexts for effective community mobilisation. AIDS Care. 2010;22 Suppl 2:1569–79. 21161761 10.1080/09540121.2010.525812

[34] Gupta GR , Parkhurst JO , Ogden JA , Aggleton P , Mahal A . Structural approaches to HIV prevention. Lancet. 2008;372(9640):764–75. 18687460 10.1016/S0140-6736(08)60887-9

[35] Tversky A , Kahneman D . Judgment under uncertainty: heuristics and biases. Science. 1974;185(4157):1124. 17835457 10.1126/science.185.4157.1124

[36] van der Straten A , Stadler J , Montgomery E , Hartmann M , Magazi B , Mathebula F , et al. Women's experiences with oral and vaginal pre‐exposure prophylaxis: the VOICE‐C qualitative study in Johannesburg, South Africa. PLoS ONE. 2014;9(2):e89118. 24586534 10.1371/journal.pone.0089118 PMC3931679

[37] Van Damme L , Corneli A , Ahmed K , Agot K , Lombaard J , Kapiga S , et al. Preexposure prophylaxis for HIV infection among African women. N Engl J Med. 2012;367:411–22. 22784040 10.1056/NEJMoa1202614 PMC3687217

[38] Corneli AM , Deese J , Wang M , Taylor D , Ahmed K , Agot K , et al. FEM‐PrEP: adherence patterns and factors associated with adherence to a daily oral study product for pre‐exposure prophylaxis. J Acquir Immune Defic Syndr. 2014;66(3):324–31. 25157647 10.1097/QAI.0000000000000158 PMC4059551

[39] Datta S , Burns J , Maughan‐Brown B , Darling M , Eyal K . Risking it all for love? Resetting beliefs about HIV risk among low‐income South African teens. J Econ Behav Organ. 2015;118:184–98.

[40] Dupas P . Do teenagers respond to HIV risk information? Evidence from a field experiment in Kenya. Am Econ J Appl Econ. 2011;3(1):1–34.22199993

[41] Jung BH , Rezk NL , Bridges AS , Corbett AH , Kashuba ADM . Simultaneous determination of 17 antiretroviral drugs in human plasma for quantitative analysis with liquid chromatography‐tandem mass spectrometry. Biomed Chromatogr. 2007;21:1095–104. 17582235 10.1002/bmc.865

[42] Marzinke MA , Breaud A , Parsons TL , Cohen MS , Piwowar‐Manning E , Eshleman SH , et al. The development and validation of a method using high resolution mass spectrometry (HRMS) for the qualitative detection of antiretroviral agents in human blood. Clin Chim Acta. 2014;433:157–68. 24661980 10.1016/j.cca.2014.03.016 PMC4039613

[43] Matta MK , Burugula L , Pilli NR , Inamadugu JK , Rao S . A novel LC‐MS/MS method for simultaneous quantification of tenofovir and lamivudine in human plasma and its application to a pharmacokinetic study. Biomed Chromatogr. 2012;26:1202–9. 22222724 10.1002/bmc.2679

[44] Eakle R , Gomez GB , Naicker N , Bothma R , Mbogua J , Cabrera Escobar MA , et al. HIV pre‐exposure prophylaxis and early antiretroviral treatment among female sex workers in South Africa: results from a prospective observational demonstration project. PLoS Med. 2017;14(11):e1002444. 29161256 10.1371/journal.pmed.1002444 PMC5697804

[45] Lester RT , Ritvo P , Mills EJ , Kariri A , Karanja S , Chung MH , et al. Effects of a mobile phone short message service on antiretroviral treatment adherence in Kenya (WelTel Kenya1): a randomised trial. Lancet. 2010;376(9755):1838–45. 21071074 10.1016/S0140-6736(10)61997-6

[46] Nigatu YT , Abera S , Mekonnen MG , Melesse WN . The role of the community conversation approach in facilitating HIV/AIDS competence and utilisation of testing services in Africa: the case of Ethiopia. Afr J AIDS Res. 2015;14(4):295–301.

[47] Thomson KA , Baeten JM , Mugo NR , Bekker L‐G , Celum CL , Heffron R . Tenofovir‐based Oral PrEP Prevents HIV Infection among Women. Curr Opin HIV AIDS. 2016;11(1):18–26. 26417954 10.1097/COH.0000000000000207 PMC4705855

[48] Westercamp N , Bailey RC . Acceptability of Male circumcision for prevention of HIV/AIDS in Sub‐Saharan Africa: a review. AIDS Behav. 2007;11(3):341–55. 17053855 10.1007/s10461-006-9169-4 PMC1847541

